# Spectral Characteristics and Displacement Sensing of U-Shaped Single-Mode–Multimode–Single-Mode Fiber Structure

**DOI:** 10.3390/s24103184

**Published:** 2024-05-17

**Authors:** Chuan Tian, Xuening Chen, Yaqi Ren, Yuxing Yang, Meng Wang, Xiaolei Bai

**Affiliations:** School of Physical Science and Technology, Inner Mongolia University, Hohhot 010021, China; tianchuan@mail.imu.edu.cn (C.T.); chenxuening@mail.imu.edu.cn (X.C.); renyaqi@mail.imu.edu.cn (Y.R.); yuxingyang@imu.edu.cn (Y.Y.)

**Keywords:** U-shaped bent fiber, SMS, micro-displacement sensor, spectral characteristics

## Abstract

The U-shaped fiber configuration represents the elementary form of micro-displacement sensing, characterized by its exceptional freedom and flexibility. The study proposes the U-shaped bent single-mode–multimode–single-mode (SMS) fiber structure that integrates the multimode interference (MMI) effect for enhanced mode dispersion and the Mach–Zönder interference (MZI) effect for spectral sensitivity improvement. The transmission spectral properties of the U-shaped SMS fiber structure with a bent radius over 1 cm are experimentally measured as the change in displacement varied within the range of 5 mm in this work. As the radius decreases, the spectrum shows redshift, which is related to the central wavelength of the peak or dips—a smaller wavelength results in a stronger redshift for the same displacement change. The average sensitivity of micro-displacement measurement within a range of 5 mm is 5.41 pm/μm, and the linearity is 99.62%. The maximum sensitivity of U-shaped SMS fiber structure is 34.46 pm/μm, with the minimum displacement change of approximately 5.804 nm. The transmission spectral properties of the U-shaped SMS fiber structure within the ranges of 50 μm, 500 μm, and 5 mm are experimentally measured in this work. This experiment observed a relatively uniform spectral drift pattern in a large range of micro-displacement sensing. The measurement range is limited by the limited spectral range of the light source and the discontinuous variation in the effective refractive index. This provides an experimental reference for further understanding the characteristics of U-shaped fiber structures and applying its application in micro-displacement sensing.

## 1. Introduction

Micro-displacement measurement plays a vital role in civil engineering, medical applications, micro-electro-mechanical systems (MEMS), automotive, geological monitoring, microfabrication, microscopy, and so on [1,2,3,4,5]. The fiber micro-displacement sensor with the properties of flexibility, high sensitivity, compact structure, and good safety has aroused the interest of researchers [6,7,8,9]. Among the many characteristics of optical fibers, the bending loss of the fiber is closely related to the bent radius. This bending spectral characteristic not only exists in quartz-clad multimode fibers, but also in plastic-clad multimode fibers, with good universality [10,11]. At the same time, the coherent characteristics of the core and cladding light in the bent fiber, the Mach–Zönder interference (MZI) effect for example, provide a critical way to measure small displacements through bent fibers [12,13,14,15,16]. The U-shaped fiber structure is the simplest bending structure with the highest degree of freedom and flexibility. Its loss and spectral properties represent the fundamental physical laws and sensing principles of bent fibers. However, due to the bending loss not having a linear variation pattern under small bent radius, simply using the bending loss of single-mode fibers is not suitable for measuring the variation in bent radius. It is necessary to use the coherent characteristics inside bent fibers for the measurement of small radius changes [17,18,19,20]. To achieve an obvious Mach-Zenhder interference effect, the bent radius needs to be less than 1 cm [21], and the sensitivity of displacement sensing is often closely related to the bent radius. In 2022, Shuying Li et al. demonstrated a U-shaped curved single-mode fiber displacement sensor with a radius of 4.4 mm, achieving a detection sensitivity of 1.28 nm/μm [21]. Mingpan Bi et al. further increased the bent radius of the U-shaped structure to 5.9 mm and the sensitivity was 59.82 pm/μm [22]. Reducing the bent radius can increase the sensitivity. However, large bending losses and fragility are invariably caused by a small bent radius. While enhancing interference wavelengths through active cavity methods is a viable approach, its complex structures and great cost limit further improvement [22,23]. A reliable way to improve micro-displacement sensing sensitivity under large-size bending situations is to consider extra interference effects. The advantages of its high modulation depth and simple structure make the multimode interference (MMI) effect the best option in bent fiber structure displacement sensing. Furthermore, the refractive index difference brought on by bending stress is particularly apparent because multimode fibers have larger core diameters, further amplifying the MZ interference effect [24,25,26,27,28,29]. In 2011, Wu et al. achieved an all-fiber displacement sensing technology relying on the single-mode–multimode–single-mode (SMS) heterojunction. This sensor with a compact structure relies solely on the MMI effect and the sensitivity of merely 5.89 pm/μm [29]. The superposition of the two interference effects in the U-shaped SMS fiber construction produces complex spectrum phenomena. In the case of millimeter-level bent radius, the spectral modulation of MMI is masked by the MZI because the extremely high high-order mode loss in multimode fibers leads to a few numbers of modes involved in multimode interference effects. Furthermore, the range of micro-displacement measurements utilizing U-shaped fiber structures is compressed by this tiny bent radius. On the contrary, if the bent radius is too large, the refractive index change caused by bending cannot be reflected in the output spectrum clearly, which will reduce the measurement sensitivity. Therefore, appropriately expanding the bent radius can balance the MZI and MMI effects in U-shaped SMS fibers, and obtain a large measurement range and high sensitivity.

In addition, various shapes of micro-displacement sensors have been designed, such as the ring-type [30], balloon-type [31,32,33,34], fluctuation meander-shaped structures [27], and special structures [35,36,37,38]. Although the complex bending structures can enhance sensitivity to a certain extent, the complex fabrication process makes the measurement results more affected by external factors, and the stability of the sensor is subject to great challenges.

In this work, a bending fiber micro-displacement sensor with a U-shaped SMS fiber structure is demonstrated. The parameters of the U-shaped SMS fiber structure are analyzed to reveal its spectral characteristics in displacement sensing. The radius of the U-shaped fiber is set to 1.2 cm to balance the MMI and MZI effects.

## 2. Experimental Setup and Sensing Principle

### 2.1. Experimental Setup

**Figure 1:**
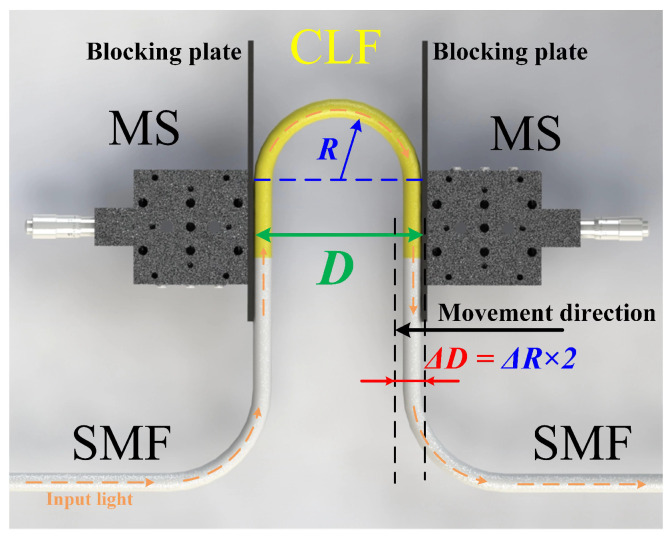
U-shaped SMS fiber structure.

**Figure 2:**
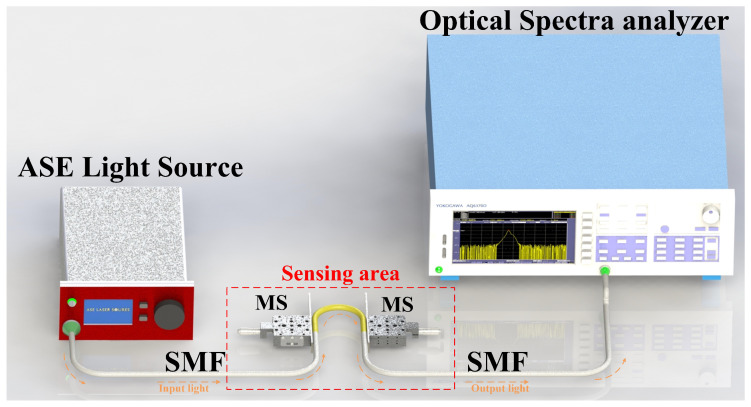
The schematic diagram of the experimental setup.

The schematic diagram of the U-shaped fiber structure is shown in Figure 1. The curved part in the middle is a segment of coreless fiber (CLF) with a diameter of 125 μm (YOFC (Wuhan, Hubei, China), CL-0/125). Both ends of the CLF are spliced with a piece of single-mode fiber (SMF, Corning Inc. (Corning, NY, USA) SMF-28e). The length of CLF is 5 cm. The bent coreless fiber (BCLF) is carefully spliced with the SMF to achieve the SMS structure. The coating layers of the BCLF are retained to reduce the transmission loss of the structure, except for approximately 5 mm near the spliced point at both ends. The multimode coreless fiber cladding material is solid quartz, and the coating layer is acrylic resin. The refractive indices of the cladding and coating are 1.495 and 1.482, respectively. The numerical aperture of the multimode fiber is 0.197. The structure is placed between two micro-stages (MS) with blocking plates fixed inside. One of them is fixed, and the other can be adjusted to fine-tune the parallel spacing (marked as *D*, *D* is the bending diameter). The ends of the multimode BCLF are securely anchored at the lower extremity of these blocking plates to ensure they remain at the same horizontal level and maintained straight near their fixed location. The radius of the U-shaped fiber structure is marked as *R* in Figure 1. The length of the BCLF exceeds the arc length corresponding to the parallel spacing to ensure the BCLF consistently maintains a stable U-shaped bending with a designated radius *R* and the symmetry of the structure. When the right MS moves a micro-displacement ΔD to the left, the change in bent radius is ΔR, and there is ΔR = ΔD/2. The experimental setup is illustrated in Figure 2. The bandwidth of the ASE light source is 50 nm, and the wavelength range is from 1520 to 1570 nm. This band is located in the lowest loss window of quartz fiber. The transmitted spectra are recorded by an optical spectrum analyzer (YOKOGAWA (Tokyo, Japan), AQ6370D).

### 2.2. Sensing Principle

The refractive index (RI) of the BCLF in a U-shaped structure is modulated by the elasto-optic effect, which is caused by transverse stress. The distribution of RI in the radial direction of the bent segment is related to the radius *R* and the stress caused by the bending. The equivalent RI ($n_{eff}$) is used to describe the influence of bending on the transverse mode distribution in the multimode fiber [39]. The $n_{eff}$ of the bent segment is expressed as follows:

(1) $$n_{eff}=n_{0}\left\{1+\frac{2-n_{0}^{2}\left[-\nu p_{11}+\left(1-\nu \right)p_{12}\right]}{2R}\right\}$$

where $n_{eff}$ is the equivalent RI of the BCLF. $n_{0}$ is the RI of the fiber without bending. ν is the Poisson ratio. $p_{11}$ and $p_{12}$ are the photo-elastic tensor in silica fiber. *R* is the bending radius, and *R* = $R_{0}$ ± ΔR. $R_{0}$ is the bending radius of the initial state. ΔR is the bending radius change. When the bending radius of the final state is smaller than that of the initial state, the sign before ΔR is negative. On the contrary, the sign is taken as the positive one, which means that the bending radius is increasing. The $n_{eff}$ increases as the bending radius of the multimode fiber decreases, and there is a similar variation pattern between the corresponding $n_{eff}$ and the bending diameter *D*.

Considering the influence of the $n_{eff}$, the transmission wavelength of MMI changes also due to the micro displacement movement. The transmission wavelength expression of the bent SMS fiber structure is expressed as [39]:

(2) $$\lambda =\frac{16\cdot n_{eff}\cdot d_{core}^{2}}{V\cdot (2V-1)\cdot L}$$

where λ is the transmission wavelength. $n_{eff}$ is the equivalent RI of the BCLF. $d_{core}$ is the core diameter of the multimode fiber, and *L* is the length of the bent segment of the multimode fiber, which is changed with the radius *R*. *V* is the number of modes in multimode fibers. Additionally, the segment of the multimode fiber without bending has the same transmission characteristics as the traditional straight MMI.

## 3. Results and Discussion

### 3.1. Simulation

**Figure 3:**
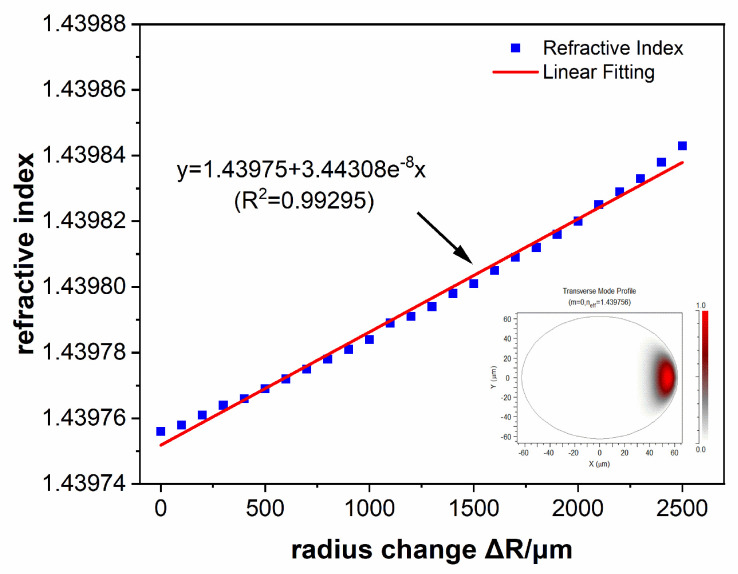
The simulation results of the $n_{eff}$ changing variation with the amount of bent radius change ΔR. The insertion is the distribution of the fundamental mode in a bent segment.

**Figure 4:**
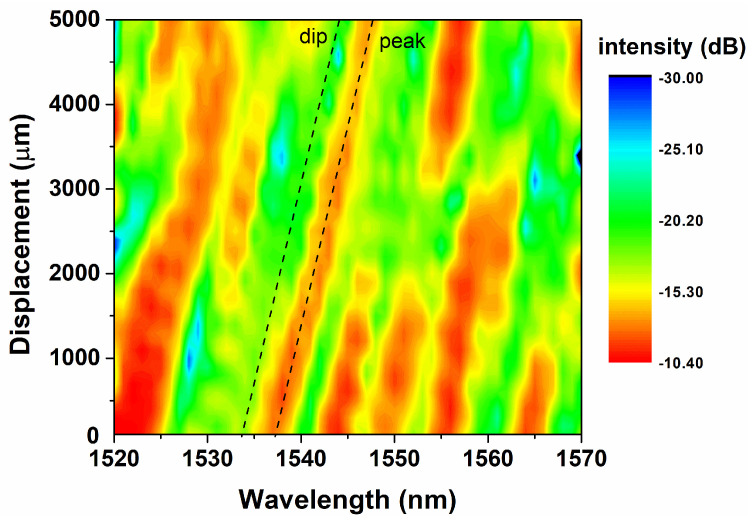
The simulation results of the evolution transmit spectra with the variation in displacement.

The spectral characteristics of the U-shaped SMS fiber structure can be simulated by the beam propagation method (BPM). The simulation results of the $n_{eff}$ changing with the change ΔR of the bent radius *R* are shown in Figure 3. Bending causes the transverse mold in multimode fibers to shift towards the side of the bent radius. The distribution of the fundamental mode in a bent segment is shown in the insertion of Figure 3. As the amount of variation in the bending radius increases, the equivalent refractive index increases. The simulation results of displacement ΔD within the range of 5 mm are shown in Figure 4. The typical marked dip or peak in the transmission spectrum has a regular red shift with the increase in displacement ΔD. The sensing information of displacement can be obtained by recording its central wavelength. There is a positive linear relationship between the absolute value of ΔR and $n_{eff}$ when the displacement bending radius decreases, which is shown in Figure 4. Conversely, the absolute value of ΔR is negatively correlated with $n_{eff}$ as R increases. Linear data fitting and the least squares are methods used to determine linear dependencies. The results of the theoretical calculations agree with the simulation trends. There is a positive linear relationship between ΔR and $n_{eff}$.

Due to the additional interference phenomena introduced in bent fibers, the transmission loss of the same mode will exhibit wavelength-dependent periodic modulation [40,41]. Because of the unstable loss, the intensity fluctuates during the spectra redshift. This leads to a jitter of characteristic wavelengths and a decrease in the recognizability of peaks or dips, which will reduce the sensitivity of displacement sensing. From Formulas (1) and (2), it can be seen that the bending state of multimode fibers influences the coupling between higher-order modes and fundamental modes in MMI. The almost constant $n_{eff}$ enables stable mode coupling in small displacement range measurements, and there are rich and distinct characteristic peaks or dips in the output spectrum. However, the loss of output light intensity fluctuates periodically when the bending radius produces large variations, and the $n_{eff}$ will also exhibit discontinuous nonlinear changes [42]. The characteristic peak intensity of the spectrum will be masked, and the characteristic wavelengths will be few and irregular. Due to the discontinuity of the effective refractive index, the upper limit of the U-shaped measurement is possible at 2.5 mm.

### 3.2. Experimental Results

**Figure 5:**
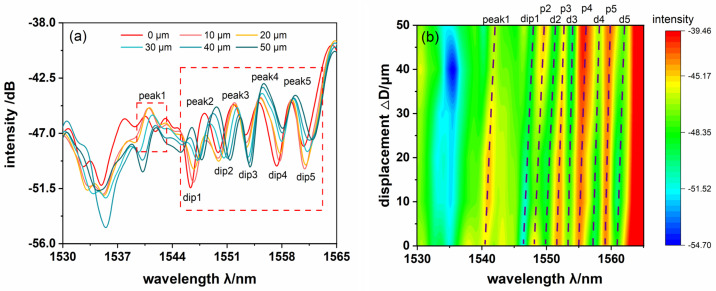
(**a**) The experimental results of the evolution transmit spectra in the range of 50 μm. (**b**) The evolution of 2D spectral intensity with displacement.

The spectral shift properties are recorded in a small displacement range of 50 μm. The comparison of the spectra is plotted in Figure 5a, and the evolution of 2D spectral intensity with displacement is shown in Figure 5b. There are many obvious peaks and dips in the output spectrum because of the multimode interference effect, which are, respectively, marked in the figure. Moreover, the loss fluctuation phenomenon is not obvious due to the small range of bent radius variation. The intensity of the spectrum remains almost the same. As the displacement increases, the overall spectrum has a regular movement toward longer wavelengths with the equivalent refractive index becomes larger (as shown in Figure 5a). The experimental results are in agreement with the trend of the results calculated by the coupled theory of (1) and (2).

**Figure 6:**
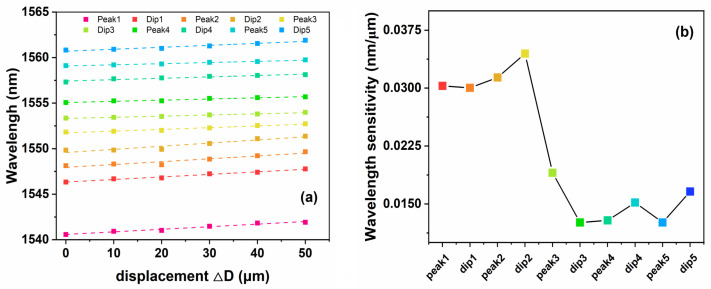
(**a**) The relationship of displacement and the wavelength of characterized peaks or dips. (**b**) The calculated sensitivity results in the range of 50 μm.

According to the movement of characteristic peaks and dips, the sensing relationship between their corresponding wavelengths and displacements is recorded as shown in Figure 6. Linear dependencies are determined using linear data fitting and least squares. The measurement results show that there is a linear relationship between the wavelength drift and the displacement. The sensitivity is calculated and plotted in Figure 6b. The maximum sensitivity of displacement is P2 (marked as peak2), with its initial center wavelength of 1548.13 nm. The sensitivity of its displacement sensing is 34.46 pm/μm, with a minimum displacement change of approximately 5.804 nm. The sensitivity is reduced to 16.6 pm/μm when the central wavelength of marked peaks or dips increases to 1559 nm. This is due to the difference in the number of modes in multimode fibers. Compared to lasers with larger central wavelengths, short-wavelength light excites more modes and is more sensitive to refractive index changes caused by bending.

Furthermore, the range of displacement variation is expanded to 500 μm to analyze the spectral property of the U-shaped SMS fiber structure within a larger displacement range. To ensure that the bending structure is a standard semicircular structure, R should be less than 1.43 cm and generally limited as aforementioned (>1 cm) [21]. We move the micro-displacement platform from its initial position (bent radius *R* = 1.2 cm) as the reference point, adjusting by the increments of 100 μm to gradually decrease *R*.

**Figure 7:**
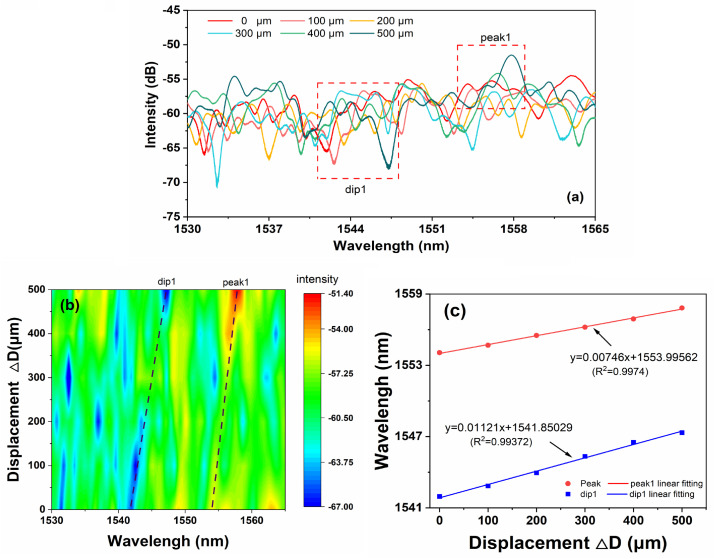
(**a**) The evolution of the spectra with the displacement in the range of 500 μm. (**b**) The 2D spectral intensity comparison. (**c**) The relationship of displacement and the characterized wavelength.

The output spectra with displacement variation are shown in Figure 7 when the length of BCLF is 5 cm. As shown in Figure 7a, when the displacement variation increased from 100 to 500 μm, the center wavelength of D1 (marked as dip1) drifted by 5.3 nm, from 1541.74 nm to 1547.04 nm, which is larger than that of P1 (marked as peak1). The wavelength of characteristic peak1 changes only 3.28 nm according to the 2D spectral intensity comparison in Figure 7b. The maximum sensitivity of displacement in 500 μm range is 11.21 pm/μm. As shown in Figure 7c, light with longer wavelengths still has relatively low displacement sensitivity, similar to small-range situations. Meanwhile, the irregular changes in bending loss caused by changes in bent radius result in a significant decrease in displacement sensitivity compared to small-scale situations.

**Figure 8:**
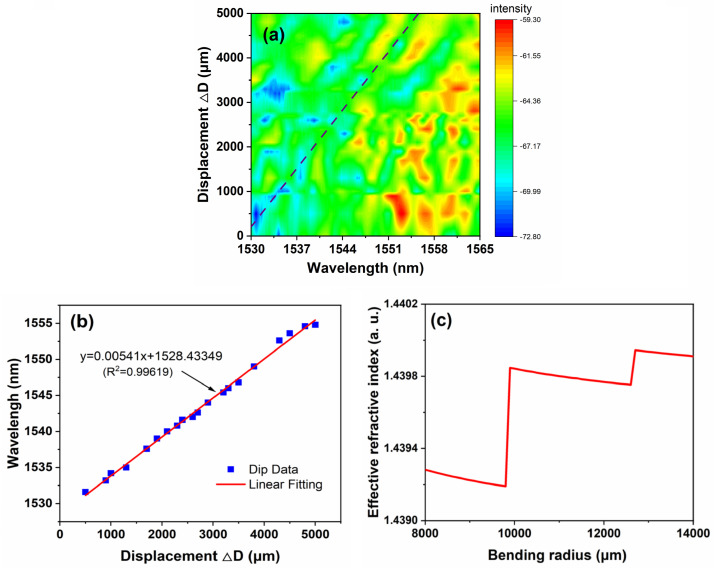
(**a**) The 2D spectral intensity comparison in the range of 5 mm. (**b**) The relationship of displacement and the characterized wavelength. (**c**) The simulated $n_{eff}$ in the range of 5 mm.

To further investigate the effective measurement range of this bent fiber structure, the displacement variation range increases to 5 mm, and the corresponding bent radius variation range is 2500 μm. The micro-displacement platform from its initial position (bent radius *R* = 1.2 cm) as the reference point. The output spectra evolve with various displacements are recorded, as shown in Figure 8a. The continuous and robust linear spectra shifting and spectral response segments could be observed, resulting in a good linear relationship between displacement and spectra. The center wavelength drifted by 23.2 nm, from 1531.6 nm to 1554.8 nm, when the displacement rose from 0 to 5 mm. The wavelength of the characterized dip is a function of the displacement variation, which is plotted in Figure 8b, and the linear fitting line of this function is also plotted. According to the measured results, the sensitivity of this displacement sensor is 5.41 pm/μm. The linearity and Pearson’s Correlation Coefficient of the sensing results in this range are high, and the R2 is about 0.99619. The experimental results above show that the structure still has good performance under large-scale measurement, especially in linearity. However, the spectral intensity exhibits significant fluctuations, which limits the further expansion of the sensing range. It is inferred that the irregular minor fluctuations of spectral response during large-range movement measurements could be caused by elastic fixation of the fixed end. The loss fluctuations caused by bending also result in irregular modulation of the output spectral intensity.

When we try to further expand the displacement sensing range, the irregularity of spectral intensity or spectral drift significantly increases, making it difficult to effectively select characteristic peaks to obtain displacement data. The spectrum exhibits obvious discontinuous jumps. At the same time, simulation analysis was conducted on the effective refractive index of the U-shaped SMS fiber structure, and the results are shown in Figure 8c.

According to Formula (1), there is a positive linear relationship between $n_{eff}$ and absolute value of ΔR. The $n_{eff}$ and λ are also positively correlated according to the Formula (2); these two relationships are consistent with the results of the simulation shown in Figure 8. In Figure 8c, the initial bending radius $R_{0}$ of the U-shaped SMS fiber structure is 12 mm. The minimum displacement bending radius is reduced to 8 mm and the maximum is increased to 14 mm (the max ΔR is 3 mm, the max ΔD is 6 mm). There are two obvious discontinuity points at the bent radius of 10,000 and 12,800 μm. It is speculated that this is one of the main reasons why a larger displacement sensing range cannot be obtained. In this work, we selected the fiber bent radius range of 10,000 to 12,500 μm, within which effective refractive linear changes are observed, making the displacement sensing results linearly measurable.

## 4. Conclusions

We theoretically calculate and experimentally verify the spectral properties of macroscopic bending U-shaped SMS structure all-fiber. The transmission spectral properties of U-shaped SMS fiber structures in the ranges of 50 μm, 500 μm and 5 mm were experimentally measured. It is demonstrated that the structure with an arc length of 5 cm processes high linearity and sensitivity at smaller bent radius. Compared to its counterparts, the designed structure is beneficial to displacement sensing with a broader range (millimeter scale). Additionally, the experimental results of displacement sensing by this structure show that the transmission spectra drift uniformly with the radius reduction in a 5 mm range of displacements. The average sensitivity obtained in this range is 5.41 pm/μm, and the linearity is 99.62%. The maximum sensitivity of 34.46 pm/μm and the minimum displacement change of approximately 5.804 nm are achieved in the range of 0 to 50 μm by optimizing the peak position of the transmission spectrum. The proposal not only offers a potential way the design and optimize U-shaped displacement sensors, but also serves as a reference for further enhancing sensor performance. An increase in the wavelength of the measured characteristic peaks results in improved linearity but reduced sensitivity. The limiting factors of the displacement sensing range is theoretically analyzed. It should be noted that, in the experiments, the elastic vibration observed in the multimode fiber, mainly stemming from the vibrations of the displacement platform, introduces minor errors in amplitude and leads to variations at specific spectral positions. Based on the analysis of the experimental results, it is speculated that a shorter wavelength may yield even greater measurement sensitivity. Future endeavors will involve exploring and refining these issues to enhance the structure’s performance and capabilities.

## Data Availability

The data presented in this study are available on request from the corresponding author.
